# How we scan cardiac anatomy and function using cardiovascular magnetic resonance: a practical video guide

**DOI:** 10.1093/ehjimp/qyaf090

**Published:** 2025-07-07

**Authors:** Jennifer Erley, Corinna Else, Wiebke Dieckhoff, Paulius Bucius, Patrick Doeblin, Collin Götze, Katja Berkmann, Christian Stehning, Sebastian Kelle

**Affiliations:** Department of Diagnostic and Interventional Radiology and Nuclear Medicine, University Medical Center Hamburg-Eppendorf, Hamburg, Germany; Department of Cardiology, Angiology, and Intensive Care Medicine, Deutsches Herzzentrum der Charité Berlin, Berlin, Germany; Department of Diagnostic and Interventional Radiology and Nuclear Medicine, University Medical Center Hamburg-Eppendorf, Hamburg, Germany; Department of Cardiology, Medical Academy, Lithuanian University of Health Sciences, Kaunas, Lithuania; Department of Cardiology, Angiology, and Intensive Care Medicine, Deutsches Herzzentrum der Charité Berlin, Berlin, Germany; DZHK (German Center for Cardiovascular Research), Partner Site Berlin; Department of Cardiology, Angiology, and Intensive Care Medicine, Deutsches Herzzentrum der Charité Berlin, Berlin, Germany; DZHK (German Center for Cardiovascular Research), Partner Site Berlin; Philips Healthcare GmbH, Hamburg, Germany; Philips Healthcare GmbH, Hamburg, Germany; Department of Cardiology, Angiology, and Intensive Care Medicine, Deutsches Herzzentrum der Charité Berlin, Berlin, Germany; DZHK (German Center for Cardiovascular Research), Partner Site Berlin

**Keywords:** strain, cardiac magnetic resonance imaging, protocol, guide, video, cardiac

## Abstract

**Aims:**

Fast Strain-encoding (fSENC) is a pulse sequence that enables the acquisition of cardiovascular magnetic resonance images within a few heartbeats and at free breathing to quantify myocardial strain, a deformation parameter of the heart muscle. Strain is gaining importance in heart failure diagnostics, but implementing fast strain-encoding into a routine magnetic resonance protocol has not been thoroughly explored from a practical viewpoint. This video manuscript aims to provide a simple guide for the acquisition of cardiovascular magnetic resonance exams in cardiac patients and to determine the scan-rescan reproducibility of segmental strain analyses.

**Methods and results:**

A volunteer was scanned for demonstration purposes on a 1.5T MRI Scanner (‘Ingenia, Philips Healthcare, Best, The Netherlands’). The acquisition of cine steady-state free precession (SSFP) and fSENC sequences is demonstrated in a step-by-step fashion, accompanied by a multilingual video tutorial and an image guide. Scan-rescan reproducibility of acquisition-based strain values was excellent between subsequent scans for segmental longitudinal (SLS) [0.93 (0.91–0.95) and circumferential strain (SCS) [0.78 (0.73–0.82) to 0.84 (0.80–0.87)], and good to excellent between scans that were interrupted by a break for SLS [0.80 (0.74–0.85) to 0.84 (0.79–0.87)] and SCS [0.57 (0.46–0.66) to 0.65 (0.56–0.77)].

**Conclusion:**

This multilingual video manuscript provides a practical guide to conducting cardiovascular magnetic resonance exams including SSFP and fSENC, useful for further quantitative analysis to grasp heart function on a global and regional basis.

## Teaser

This manuscript has the aim to guide through a cardiac magnetic resonance imaging protocol to assess cardiac anatomy and function, including the acquisition of cine-balanced steady-state free precession images for evaluation of conventional functional and volumetric parameters, and fast strain-encoded images for myocardial strain (deformation) analysis.

## Graphical Abstract

**Graphical Abstract:**
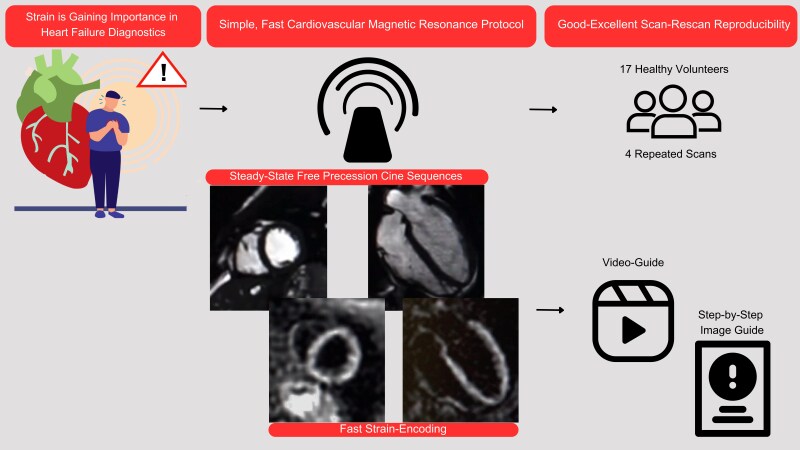
Video manuscript to provide a simple guide for a cardiac magnetic resonance (CMR) imaging protocol to assess cardiac anatomy and function, including the acquisition of cine-balanced steady-state free precession (SSFP) images for evaluation of conventional functional and volumetric parameters, and fast strain-encoded (fSENC) images for myocardial strain (deformation) analysis.

## Introduction

Heart failure is one of the most common causes of hospitalization and death, with 1–2% of the population in the Western world being affected.^1^ It accounts for more primary care consultations than angina due to the drastic decrease in quality of life caused by the associated symptoms.^2,3^ The pathologies resulting in heart failure are diverse and at times difficult to diagnose. To effectively treat and prevent heart failure, it is urgent to notice the associated decline in myocardial function at an early and sometimes asymptomatic stage.

Cardiac magnetic resonance imaging (CMR) is the gold standard imaging procedure for the evaluation of cardiac morphology and function.^4^ Currently, patients are categorized into heart failure stages using a parameter called ejection fraction (EF),^5^ which can be determined from conventionally acquired cine steady-state free precession (SSFP) CMR sequences. EF is calculated as the ratio of the stroke volume divided by the end-diastolic volume, representing the systolic function of the heart.^5^ However, half of the patients suffering from heart failure do not show any reduction in EF,^5^ possibly because EF is influenced by different factors like loading conditions and cardiac remodelling, leading to reduced or increased end-diastolic volume.^6^ Since the EF does not seem to fully embrace the underlying mechanisms and the according stages of heart failure, further parameters were studied to gain additional information on myocardial contractility beyond the EF.

In that sense, strain-encoding (SENC) was developed as a new pulse sequence by Osman et al.^7^ to quantify a different parameter of myocardial contractility, myocardial strain. Strain determines the change in length of the myocardial muscle fibres from end-diastole to end-systole. It is expressed in % and has a negative value during systole, as the muscle fibres shorten during contraction.^8^ Strain can be determined in a longitudinal and circumferential orientation using SENC, in line with the different orientations of the muscle fibres^9^ (radial strain cannot be assessed with this pulse sequence). Furthermore, it can be determined on a regional basis as well, whereas the EF can only be determined globally. Fast strain-encoding (fSENC) was introduced as a further development of SENC, allowing SENC scans to be acquired within a few heartbeats only.^10^

Despite its informative value, the use of CMR in clinical routine is still restricted due to a lack of acquisition and analysis skills^11^ and the perception that CMR exams are too long with strenuous breath holds. There are official guidelines regarding image acquisition^12^ and post-processing of CMR exams.^13^ However, these guidelines could be difficult to comprehend for personnel in training who are inexperienced with CMR, and they do not include novel imaging sequences, such as fSENC. Thus, it is not always clear how the strain analysis is best integrated into the CMR protocol. Hence, this multilingual video protocol was filmed to explain how a short and practical CMR exam of a cardiac patient could be conducted without the administration of contrast agent using a Philips 1.5 T scanner (*Ingenia*).

## Methods

The following protocol is a step-by-step guide to scanning cardiac patients using CMR to determine important biventricular volumetric and functional parameters such as mass, volumes, EF, and strain. The steps are also explained in the corresponding videos in English and German (see Supplementary data online, *Videos S1* and *S2*). Furthermore, the steps and image explanations can be found in an additional PowerPoint image guide (see Supplementary material). The technical parameters of the image sequences are listed in *Table 1*. The image acquisition steps vary if scanners from different vendors are used. This protocol does not replace official training. Furthermore, depending on the reason for cardiac imaging (e.g. screening for inflammatory or infiltrative disease), further scanning sequences might be necessary (such as mapping or late gadolinium enhancement), which would go beyond the scope to explain in this context. All scans should be conducted under medical supervision.

**Table 1:** Technical parameters of the cine-SSFP and fSENC sequences

	Cine-SSFP	fSENC
FOV (mm^2^)	470 × 420	256 × 256
Slice thickness (mm)	8	10
Acquired voxel size (mm^3^)	1,83 × 1.83 × 8.00	4 × 4 × 10
Reconstructed image size (mm^3^)	1.48 × 1.48 × 8.00	1 × 1 × 10
Acquisition scheme	Cartesian, SENSE = 2	Single shot spiral
Acquisition time	9 s	1 s
Flip angle (°)	60	30
Effective echo time (ms)	1.53	0.7
Repetition time (ms)	3.1	12
Temporal resolution (ms)	30	36
Number of acquired heart phases	30	22
Spectrally selective fat suppression (SPIR)	No	Yes
Total acquisition time per slice (s)	Min. 9	<1

### Patient preparation

Inform the patient about the exam, perform an essential technical anamnesis to understand if the patient can enter the magnet room, and ask if the patient consents to the scan. Establish if she/he has metallic objects in her/his body and, in case of MR-conditional devices, make sure you have the data needed to set the transmission pulses. Position the patient on the bed, so that the heart can be as close as possible to the isocenter, ensuring that the patient is as comfortable as possible (providing, if necessary, the use of pillows or pads). If it is necessary, position the breathing monitor and check that it is working properly. Connect the electrocardiogram electrodes, ensuring a clean and artefact-free signal. Connect the body array. Advise the patient to lie still for the entire duration of the examination. Explain the breathing commands to the patient and show the patient how to use the device (buzzer) to communicate with the operator and interrupt the exam. Do a test of inhaling, exhaling and holding the breath for a time similar to that used during the scan. Allow the patient to test the buzzer. Comfortably position the headset on the patient for noise reduction. Finally position and connect the coil, set the centre of the scan, and proceed with the insertion of the patient into the gantry.

### Cine-SSFP images

The following scans can be used to calculate mass, end-diastolic and end-systolic volumes, stroke volumes, and EF with any given software.

Part 1: Survey = The session will start by clicking the green ‘Start Scan button’ on the scanning console. In case another box appears, asking to ‘allow all scans, which require Whole Body SAR >2 W/kg’, click ‘Confirm and Start’ if the patient has no magnetic implants or ‘MRI-safe’ magnetic implants (considering the scanners’ field strength). If the patient has magnetic implants considered as ‘MRI-conditional’, the SAR might need to be adjusted according to the specifications of the manufacturer. Fast single-shot protocols are used for the survey.

#### Starting the scan during breath-hold

Using the microphone, ask the patient to breath in and check that the breathing graph goes up. When the breathing graph reaches the upper point, ask the patient to breath out and control that the breathing graph reaches the lowest point. At this point, ask the patient to hold his breath. While the patient is holding his breath, click ‘OK’ to start the scan (or ‘Continue Scan’ for subsequent sequences). After the scan has finished, ask the patient to continue breathing normally. It is important that the patient does not inhale and exhale too deeply and keeps the same, calm breathing rate during the examination to reduce motion artefacts.

After the survey is scanned, the image in the right-hand corner shows the current slice scanned. The survey will be shown in a transversal, coronal, and sagittal view in the three upper frames. Using the survey, check that the heart is positioned centrally in the magnet and if the correct coils are chosen.

Part 2: Right anterior oblique (RAO) = Double-click on the ‘RAO’-sequence (names may vary in your institution) in the left-hand panel to start planning. For scanning of further orientations, it is important to establish a unique longitudinal axis of the left ventricle. The longitudinal axis of the left ventricle is defined as the line connecting the centre of the mitral valve annulus to the apex of the left ventricle, identified at end-diastole (the phase of maximal ventricular filling, just before mitral valve closure). The orange stack is therefore positioned through the apex of the left ventricle and the centre of the mitral valve. It is possible to find the structures in the transversal survey images.

#### Adjusting the ‘shim-box’

The ‘shim box’ is a green box that needs to be adjusted to the size of the heart in all sequences. Ensure, that the ‘shim box’ is adapted to the size of the heart in all survey images. Often, the ‘shim-box’ is then automatically adopted for subsequent scans, and no further positioning is needed after the survey. However, adjusting the volume of the ‘shim-box’ often helps in case of flow artefacts.

Click ‘Accept’. A small box will appear when the scanner finishes loading. Start the scan, as described before. Drag the RAO image into the left-hand image frame, replacing the transversal survey image.

Part 3: Pseudo 4 chamber (p4CH) = Double-click on ‘p4CH’-sequence in the left-hand panel. Place the orange stack in the correct position on the RAO-sequence (reflecting the longitudinal axis), through the LV-apex and the centre of the mitral valve. Adjust the shim-box, if needed. Click ‘Accept’ when ready. Start the scan, as previously described. Drag the newly acquired p4CH view into the left-hand frame for further planning.

Part 4: Whole heart short axis view (sSA rest wh) = Double-click on ‘sSA rest wh’-sequence in the left-hand panel. On the p4ch view, insert a helping line by clicking the right mouse button on the image. Choose ‘Measurement’ and then ‘Line’ using the left mouse button. Plan the orientation line through the apex of the heart and the centre of the mitral valve leaflets. Adjust the orange stack perpendicularly to the orientation line, covering the whole ventricle. If the number of slices does not fit the entire ventricle, it can be increased by clicking on ‘Geometry’ and then ‘Slices’. Start the scan, as described before in the section "Starting the scan during breath-hold". Adjust the shim-box, if needed, and start the scan. It will take three to six breath-holds until all images are acquired. The progress can be seen on the blue bar of the left-hand side panel. Once one scan is finished, repeat the breath-hold, and click ‘Continue Scan’ again. Drag the newly acquired sSA rest wh-images into the left-hand frame of the image panel when finished.

Part 5: 4-chamber view (4CH) = Double-click on ‘4CH’-sequence in the left-hand panel. Using the SAXwh scan, plan the orange stack through the papillary muscles, directly intersecting the peak of the right ventricle. Adjust the shim-box, if needed. Using the coronal survey, check to ensure that the plane runs below the aortic root. Start the scan, as described before. Then drag the 4CH view into the left-hand frame.

It is also possible to plan the 4CH view using the 3-point-plan scan. To do so, place the points in the following landmarks in the SA view: the apex, the centre of the left ventricle (preferably in a basal view), and the pericardiophrenic angle of the right ventricle (preferably midventricular). There are many ways to reach these points, but it is important that this condition is achieved.

In cases of anatomical variations—such as a low (caudal) origin of the ascending aorta causing partial intrusion of the left ventricular outflow tract (LVOT) into the 4CH view—it may be necessary to slightly adjust the angulation of the imaging plane. To do so, cranially tilt the basal end of the slice, so that the plane cuts more posteriorly through the mitral valve (seen in the basal SA slice). Using the coronal survey, check to ensure that the plane runs below the aortic root.

Part 6: 2-chamber view (2CH) = Double-click on ‘2CH’-sequence in the left-hand panel. On the 4CH view, plan the stack parallel to the septum through the apex and the centre of the mitral valve leaflets. It does not need to be parallel to the septum. Adjust the shim-box, if needed. Start the scan, as described.

Part 7: 3-chamber view (3CH) = Double-click on ‘3CH’-sequence in the left-hand panel. Select the ‘3-point plan tool’ of the upper panel. Place the first point in the centre of the mitral valve (in the 4CH view), the second point in the apex (also in the 4CH view), and the third one in the LVOT of the coronal survey stack. Then select the ‘Compute Plane’-tool from the upper panel to show the orange stack. Control the correct position of the stack in the basal short axis-slice, showing the LVOT. The stack should be placed directly inside and parallel to the LVOT. The ‘3-point tool’ is not available for all vendors. In case it is not available, the 3CH can be planned by first positioning the stack in a basal SA-view from the centre of left ventricle to the centre of aortic valve. After this, using the 2CH view, ensure that the stack passes through the apex. You can check this last point using an apical SA-view. Adjust the shim-box, if needed. Start the scan, as described.

### fSENC-scans for strain analysis

The following scans can be analysed using the Myostrain software (Myostrain, Myocardial Solutions, Inc., Morrisville, North Carolina, USA).

Part 1: fSENC apical short-axis view (SA a) = compared with the cine-SAX view, the fSENC SAX scans are only performed at three slices: basal, midventricular, and apical. These planes are defined along the longitudinal axis of the left ventricle (from 0% at height of the mitral valve annulus to 100% at height of the apex). The apical slice should be positioned at 75–85% of the longitudinal axis, directly above the apex. The left ventricular cavity should still be visible, but papillary muscles should already be absent. Double-click on the ‘fSENC SA a’-sequence (names may vary in your institution) to select the view from the left-hand panel. Drag the ‘sSA rest wh’ images into the left-hand frame and select an apical view. Using the ‘3-point plan tool’ as described for the 3CH view, copy the geometry from this view (placing the three points around the image to copy). A white box, called the ‘SENC-box’, will appear. Position this box with the heart in the centre. Now drag the ‘4CH’ view into the left-hand frame and the ‘2CH’ view into the middle frame to control the stack in both views. It is important to adjust the stack in both views perpendicularly to the myocardium. As the left ventricular myocardial walls converge towards the apex, it is not possible to obtain the stack exactly perpendicular to both walls. However, it is important that the stack is perpendicular to longitudinal axis. Click ‘Accept’ and ‘Continue Scan’ without breathing commands.

Part 2: fSENC midventricular short-axis view (SA mid) = The midventricular slice should be planned at 50% of the longitudinal axis, corresponding to the level of the papillary muscles. Double-click on the ‘fSENC SA mid’-sequence to select the view from the left-hand panel. As described in point 2.1, copy the geometry of a midventricular slice of the whole-heart cine-SSFP scan using the ‘3-point plan tool’. Control the white ‘SENC-box’ to ensure, that the heart is placed in the centre of the box. Using the 4CH and 2CH views, ensure that the stack is placed perpendicular to the myocardium. Continue scanning at free breathing.

Part 3: fSENC basal short-axis view (SA bas) = The basal slice should be planned at 15–25% of the longitudinal axis, including the basal portions of the papillary muscles. Care should be taken to exclude the mitral valve leaflets and the LVOT. Double-click ‘fSENC SA bas’-sequence to select the view from the left-hand panel. Copy a basal slice of the ‘sSA rest wh’ scan using the ‘3-point plan tool’. Again, control the position of the ‘SENC-box’ and ensure, that the stack is oriented perpendicular to the myocardium in the 4CH and 2CH view. Continue scanning at free breathing.

Part 4: fSENC 4CH view = Select ‘fSENC rest 4ch’-sequence from the left-hand panel. Copy the geometry from the cine-SSFP 4CH scan by dragging the cine-4CH view into the left image frame. Ensure that the heart is located directly in the centre of the white ‘SENC-box’. Continue scanning at free breathing.

Part 5: fSENC 2CH view = Select ‘fSENC rest 2ch’-sequence from the left-hand panel. Copy the geometry from the cine-SSFP 2CH scan by dragging the cine-2CH view into an image frame. Ensure that the heart is located directly in the centre of the ‘SENC-box’. Continue scanning at free breathing.

Part 6: fSENC 3CH view = Select ‘fSENC rest 3ch’-sequence from the left-hand panel. Copy the geometry from the cine-SSFP 3CH scan by dragging the cine-3CH view into an image frame. Make sure, that the heart is located directly in the centre of the ‘SENC-box’. Continue scanning at free breathing.

### Quality check

Play through all scans after dragging them into the image frames. Verify the correct position of the heart in the image centre. Also, verify the image quality and the absence of artefacts. If there are strong flow artefacts in the images but the angulation is correct, it is helpful to reduce the shim volume slightly. This can reduce flow artefacts.

In case a scan has insufficient image quality (e.g. due to artefacts), repeat the scan. In case of disturbing artefacts, simply rotate the stack to minimize the artefacts and repeat the scan. This is shown in the video using the fSENC rest 4CH scan. Open the scan (double-click on the left-hand panel). Rotate the stack (orange square) and repeat the scan.

After contouring and analysing the fSENC images, a bull’s eye plot helps to understand the regions of reduced strain. This is especially interesting to further differentiate between patients with underlying inflammatory or rheumatoid diseases and patients with diseases affecting only a specific territory of the heart (such as coronary artery disease). By means of the above-mentioned protocol, seventeen healthy volunteers (age 24 ± 5 years, 53% females) were scanned at the German Heart Institute Berlin using a Philips 1.5T scanner (Ingenia, Philips Healthcare, Best, The Netherlands). The volunteers were scanned four times: two subsequent scans, a 15-minute break and further two subsequent scans. The strain analysis was conducted using dedicated software (‘Myocardial Solutions, Inc., Morrisville, North Carolina, USA’) on a global and segmental basis, as recommended by guidelines of the American Heart Association.^14^ Endo- and epicardial contours were manually drawn at end-systole with excluding the papillary muscles and trabeculae, as previously described.^15^ Scan-rescan variability was determined using intraclass correlation coefficients (ICC) (two-way mixed, absolute agreement). The following cut-off values were used for interpretation of ICC values: <0.40 = poor, 0.40–0.59 = fair, 0.60–0.74 = good, and 0.75–1.00 = excellent.^16^ Statistical analyses were conducted using SPSS (‘Version 28.0.1.1, IBM, Armonk, NY’). The study regarding reproducibility was approved by the Ethics Committee of the Charité-University-Medicine in Berlin and complied with the Declaration of Helsinki. It was registered at the German Register for Clinical Studies (DRKS) (resgistration number: 00013253) and the World Health Organization (WHO) (UTN: U1111-1207-5874). The results on scan-rescan reproducibility of the global strain analysis were previously published by Erley et al.^15^

## Results

*Table 2* shows the results of the scan-rescan reproducibility analysis regarding pooled segmental longitudinal (SLS) and circumferential strain (SCS). Overall, the scan-rescan reproducibility was good to excellent. The highest reproducibility was found between subsequent scans (e.g. scan 1 vs. 2 and scan 3 vs. 4) for both SLS (ICC = 0.93 [95% confidence interval 0.91–0.95 between scan 1 and 2 and 0.93 [0.91–0.95] between scan 3 and 4) and SCS [ICC = 0.84 (0.80–0.87) between scan 1 and 2 and 0.78 (0.73–0.82) between Scan 3 and 4]. In comparison, scan-rescan reproducibility was marginally lower for scans that were interrupted by a 15-minute break, with ICC values ranging from 0.84 (0.79–0.87) for SLS between Scan 1 and 3 to 0.57 (0.46–0.66) for SCS between Scan 1 and 4. Furthermore, scan-reducibility was overall better for SLS than for SCS.

**Table 2:** Scan-rescan variability of SLS and SCS measurements using fSENC

	ICC	95% CI
SLS		
Scan 1 vs. 2	0.93	0.91–0.95
Scan 2 vs. 3	0.81	0.75–0.85
Scan 3 vs. 4	0.93	0.91–0.95
Scan 1 vs. 3	0.84	0.79–0.87
Scan 1 vs. 4	0.83	0.79–0.87
Scan 2 vs. 4	0.80	0.74–0.85
SCS		
Scan 1 vs. 2	0.84	0.80–0.87
Scan 2 vs. 3	0.65	0.56–0.77
Scan 3 vs. 4	0.78	0.73–0.82
Scan 1 vs. 4	0.57	0.46–0.66
Scan 2 vs. 4	0.60	0.50–0.68

SLS, segmental longitudinal strain; SCS, segmental circumferential strain; ICC, intraclass correlation coefficient; CI, confidence interval.

## Discussion

For the correct diagnosis of cardiac patients and especially for the classification of heart failure, it is important to use non-invasive, objective, and reliable methods to determine left ventricular function. Different cardiac imaging modalities are available in most centres today, each with advantages and drawbacks. The first modality of choice to visualize cardiac structure and function is Echocardiography. Nevertheless, echocardiographic image quality might not be sufficient in certain patients to allow reliable diagnostics, for example in obese patients with insufficient diagnostic windows, and in patients with cardiac arrhythmias or the inability to hold their breath. Especially in these patients, CMR would serve as an excellent diagnostic tool.^10^ Moreover, CMR is the gold standard for evaluating cardiac morphology and function,^4^ and has the unique ability to determine myocardial oedema, siderosis, perfusion, and fibrosis.^17^ Despite these capabilities, its use is often restricted in clinical routine due to many factors, including missing skills to routinely perform and interpret CMR exams, seemingly high costs, long examination times, and the use of potentially harmful contrast agent.^11^ This video article demonstrates how a simple and quick CMR exam can be performed in cardiac patients, including explanations on scanning of conventional cine-SSFP and fSENC images.

The conventionally acquired cine-SSFP sequences allow for reliable monitoring of mass and volumes, as well as analysis of EF. These parameters provide an important overview of the haemodynamics of the patient's heart and should be determined for every CMR exam. Regarding strain analysis, a variety of techniques are available using CMR, such as tagging, feature tracking (FT), displacement-encoding, and SENC/fSENC, all with different advantages and drawbacks.^8^ Tagging is the oldest technique with many validation studies, thus considered the ‘gold standard’ for strain quantification.^18^ However, tagging images suffer from low spatial and temporal resolution, as well as tag fading, reducing its accuracy, especially with thin cardiac walls.^19^ FT can be performed on cine-SSFP images without the need for additional image acquisition. Moreover, FT also allows the assessment of radial strain. On the other hand, FT has shown to be less reproducible than acquisition-based techniques (such as SENC and fSENC) regarding segmental strain.^20^

The use of fSENC requires additional scanning, but the extremely short acquisition time of a few heartbeats is shorter compared with tagging, and does not relevantly prolong the examination time (as seen in the video).^21^ Also, fSENC provides a better temporal resolution than tagging and the cine images used for FT.^9^ It is recommended to train the technologists, as well as the analysts before using fSENC in clinical routine. However, the effects of the short training can be seen by the excellent reliability determined between observers with different levels of experience.^22^ Also, as indicated by the good to excellent ICC values in this study, our representative results show that fSENC can be used to reproducibly assess segmental left ventricular strain. In line with these results, a previous analysis by Bucius *et al*.^20^ found that fSENC, scanned using the above-mentioned protocol, outperforms FT regarding the intra-observer and scan-rescan reproducibility of segmental strain analyses. This is crucial to reliably introduce segmental strain into clinical routine. As an example, due to the high reproducibility on a segmental basis, fSENC could be able to reliably differentiate between a subendocardial and transmural infarction, which is currently only possible after contrast agent administration.^23^ Furthermore, intra-observer agreement was excellent when examining volunteers and heart failure patients using the above-mentioned protocol.^20^ This is important to note as reproducibility is crucial to standardize strain measurements. No breathing commands are needed, making fSENC especially attractive to scan uncooperative patients, as well as children and patients with pulmonary and myocardial diseases.^10^ Also, fSENC strain measurements correlate well with other techniques, such as FT and tagging.^20,24^ The fSENC pulse sequence is vendor-independent (currently available as a product with Philips scanners and as a patch with other vendors) and previous data have shown that fSENC strain measurements acquired at different scanners show good inter-vendor agreement.^15^ Both FT and SENC/fSENC allow for analysis of right ventricular strain,^25^ but this article focuses on strain analysis of the left ventricle, as only left ventricular strain is recommended for clinical use in guidelines.^26^

Regarding the clinical applications of fSENC, Lapinskas et al. previously published the results of scanning volunteers, heart failure patients with preserved EF (HFpEF) and heart failure patients with reduced EF (HFrEF), also using the above explained scanning protocol.^22^ Strain values were significantly lower in HFrEF patients and even HFpEF patients compared with healthy volunteers.^22^ Another previous study by Korosoglou et al. has shown that fSENC enables identification of subclinical cardiac dysfunction in patients with a high risk of heart failure and the associated complications.^27^ These results demonstrate that strain, determined using fSENC, provides additional information beyond EF, as it has the ability to differentiate between healthy subjects and HFpEF patients with physiological EF.

## Conclusion

In summary, the above-described protocol can be used to diagnose and evaluate cardiac patients, especially potential heart failure patients at a stage where conventionally measured parameters, such as EF, might still be preserved.^22^ In this video manuscript, we explained the principles of scanning conventional cine-SSFP sequences and fSENC images. Furthermore, we showed that fSENC can be easily integrated into a standard CMR protocol and allows for the fast acquisition of images for strain analysis with high scan-rescan, intra-observer, and inter-observer reproducibility.

## Supplementary Material

qyaf090_Supplementary_Data

## Data Availability

The data underlying this article will be shared on reasonable request to the corresponding author.

## Lead author biography

Jennifer Erley is a German scientist specialized in the fields of cardiovascular magnetic resonance imaging and coronary computed tomography angiography. Her research interests are strain imaging, cardiomyopathies, and coronary artery disease. She is currently a fifth-year radiology resident at University Medical Center Hamburg-Eppendorf. Moreover, she is a board member of the ‘Cardiovascular Diagnostics’ working group of the German Radiological Society.
