# Supplementary material for: How we scan cardiac anatomy and function using cardiovascular magnetic resonance: a practical video guide
Source: Eur Heart J Imaging Methods Pract. 2025 Jul 7;3(2):qyaf090. doi: 10.1093/ehjimp/qyaf090 (PMC12287925; doi:10.1093/ehjimp/qyaf090)
Supplement: qyaf090_Supplementary_Data [file qyaf090_supplementary_data.zip › Step-by-step-guide-Erley-EHJ-IMP-2025.pdf]

DEUTSCHES HERZZENTRUM
DER CHARITÉ

HAMBURG

# How we scan cardiac anatomy and function using cardiovascular magnetic resonance: a practical video guide

Cine-SSFP sequences + fSENC sequences

Jennifer Erley MD[1], Corinna Else (RT)[2], Wiebke Dieckhoff (RT)[1], Paulius Bucius MD[3], Patrick Doeblin MD[2,4], Collin Götze[2,4], Katja Berkmann[5], Christian Stehning PhD[5] and Sebastian Kelle MD[2,4]

[1]Department of Diagnostic and Interventional Radiology and Nuclear Medicine, University Medical Center Hamburg-Eppendorf, Hamburg, Germany
[2]Department of Cardiology, Angiology, and Intesive Care Medicine, Deutsches Herzzentrum der Charité Berlin, Berlin, Germany
[3]Department of Cardiology, Medical Academy, Lithuanian University of Health Sciences, Kaunas, Lithuania
[4]DZHK (German Center for Cardiovascular Research), Partner Site Berlin
[5]Philips Healthcare GmbH, Hamburg, Germany

CHARITÉ

MRI-CORE-LAB
BERLIN

# Table of Contents

1. Cine-SSFP-images
   1.1 Survey
   1.2 Right Anterior Oblique (RAO) sequence
   1.3 Pseudo 4 chamber (p4CH) sequence
   1.4 Whole-heart short axis (sSA rest wh) sequence
   1.5 4-chamber (4CH) sequence
   1.6 2-chamber (2CH) sequence
   1.7 3-chamber (3CH) sequence
2. fSENC
   2.1 fSENC short-axis apical sequence
   2.2 fSENC short-axis midventricular sequence
   2.3 fSENC short-axis basal sequence
   2.4 fSENC 4-chamber sequence
   2.5 fSENC 2-chamber sequence
   1.6 fSENC 3-chamber sequence
3. Quality check

# 1.1 Survey

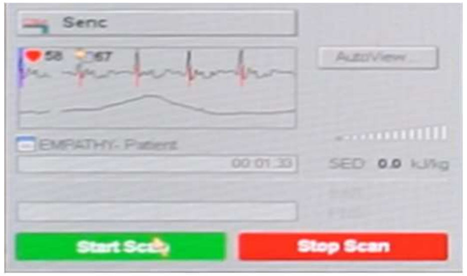


1. Click the green “Start Scan” button to start the session.

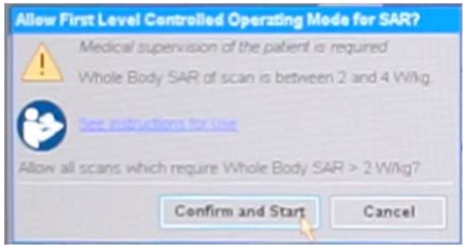


2. Click „Confirm and Start“.

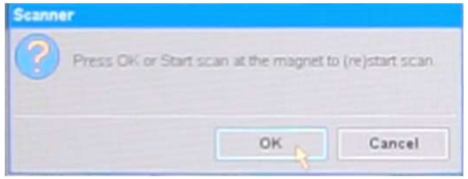


3. Click „OK“ while the patient holds his breath to start the scan.

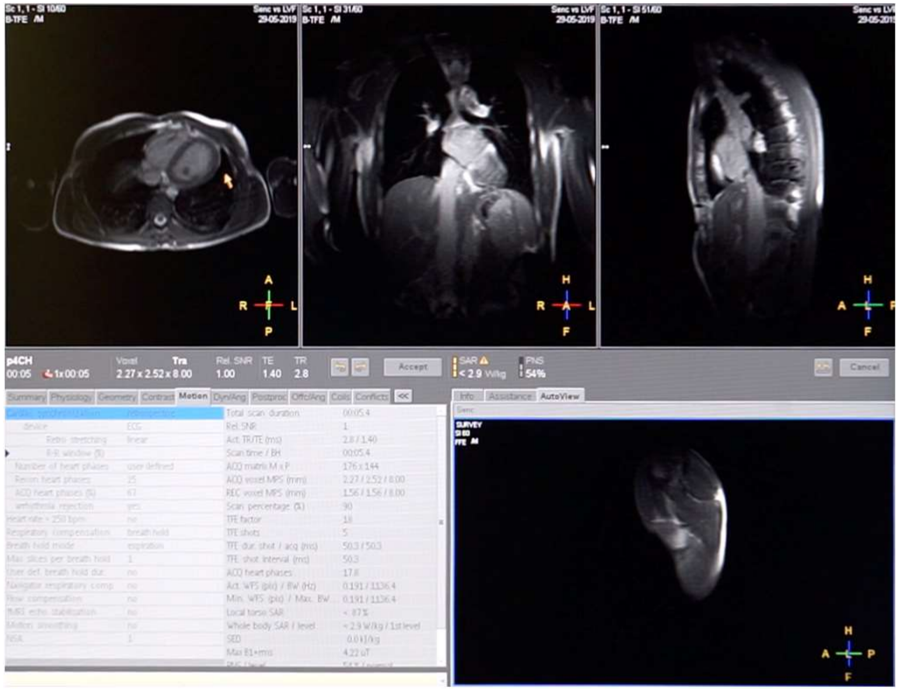


3. After scanning is performed, the survey is shown in the transversal (left), coronary (middle) and sagittal (right) views.

# 1.2 Right Anterior Oblique (RAO)

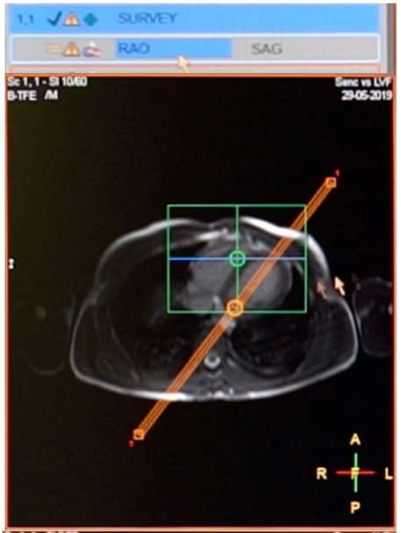


1. Double-click “RAO”-sequence on the left-sided panel to start planning.

2. Move the orange stack through the apex of the left ventricle and the center of the mitral valve.

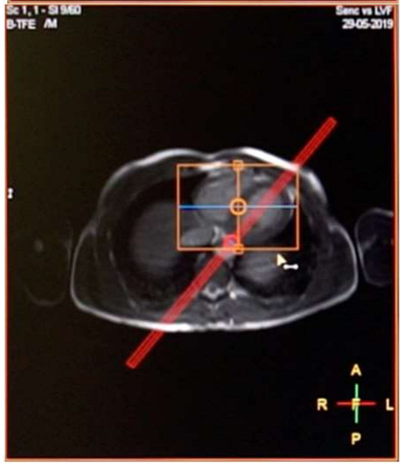

3. Adjust the ”shim-box” to the size of the heart in all views.

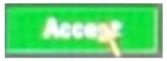


4. Click “Accept” when finished with planning.

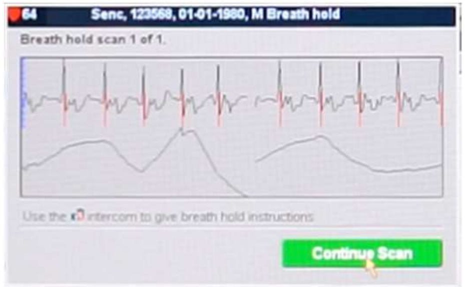


5. A new box will appear, as shown on the left. Click “Continue Scan” to start scanning while the patient holds his breath.

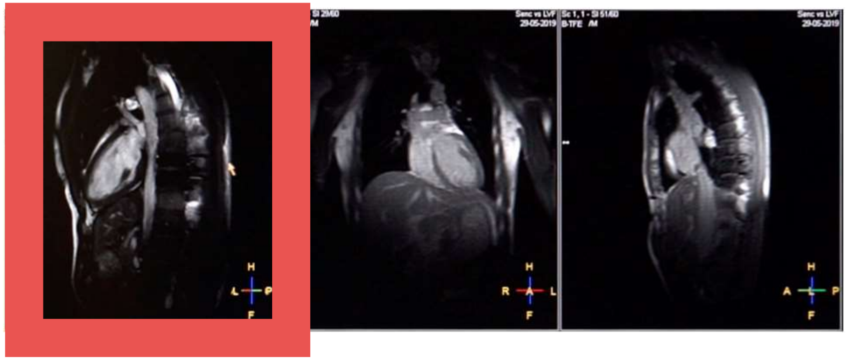

6. When finished, drag the newly acquired “RAO” image into the left-hand frame of the image panel (red).

# 1.3 Pseudo 4 Chamber (p4Ch)

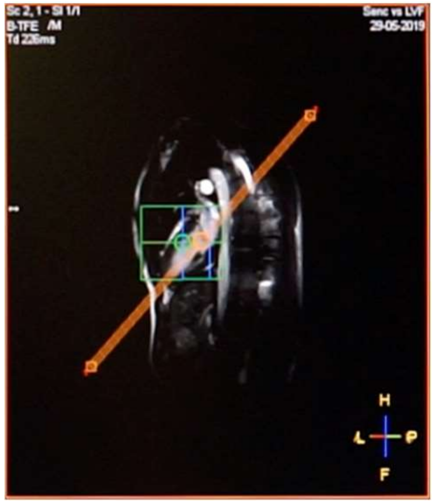

1. Double-click “p4CH”-sequence on the left-sided panel to start planning (shown as step 1 page 4). Adjust the orange reference line on the RAO-view, pointing through the LV-apex and the center of the mitral valve. Ensure the “shim-box” fits the heart.

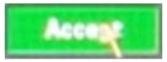


2. Click “Accept” when finished with planning.

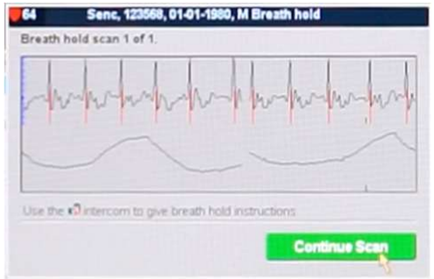


3. While the patient holds his breath, click “Continue Scan”.

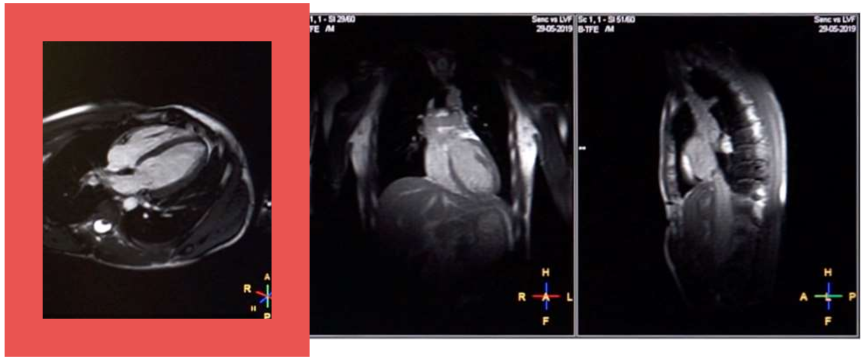

4. Drag the p4CH scan into the left frame of the panel to continue planning.

# 1.4 Whole-heart short axis view (sSA rest wh)

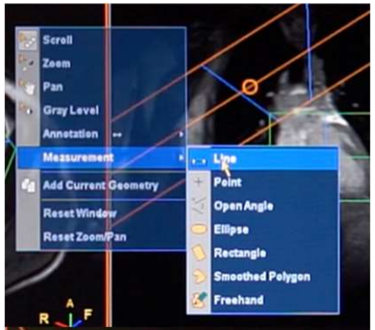


1. Double-click “sSA rest wh”-sequence on the left-hand panel. Insert an orientation line: right-click on the p4CH image → choose “Measurements” and “Line”.

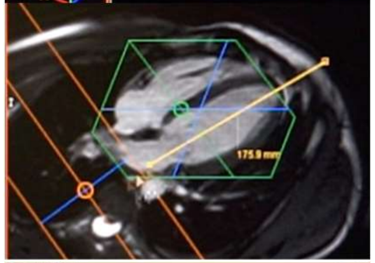

2. Place the orientation line through the apex and the center of the mitral valve leaflets.

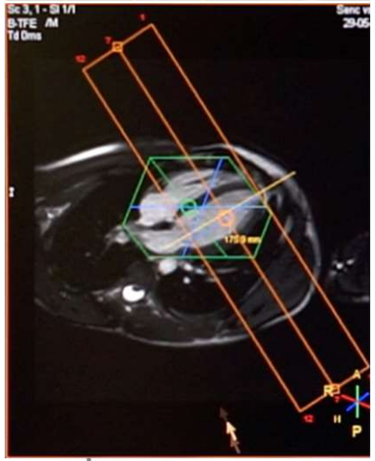

3. Place the orange stack perpendicular to the orientation line. Ensure, that it covers the whole ventricle.

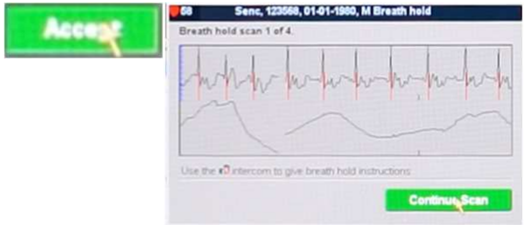


4. Start the scan as described before, while the patient holds his breath.

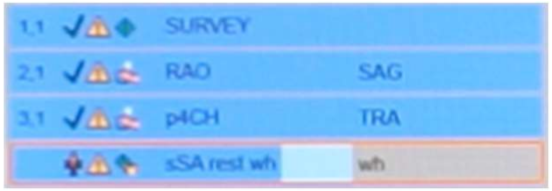


5. It will take up to 3-6 scans to acquire the whole heart. The progress can be seen on the blue bar of the left-hand panel.

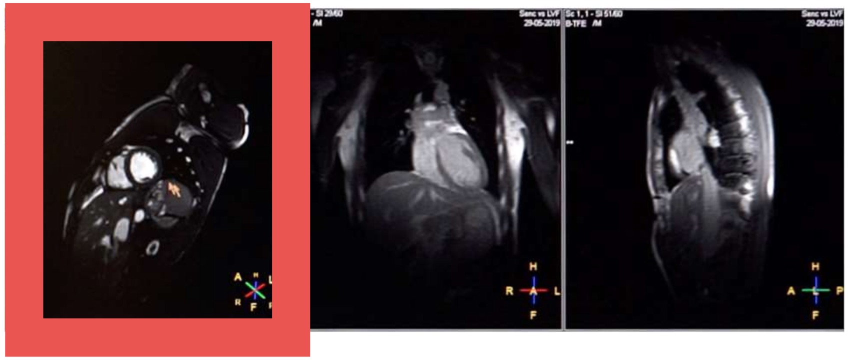

6. After scanning is finished, drag the sSA rest wh into the left-hand frame.

# 1.5 4-chamber view (4CH)

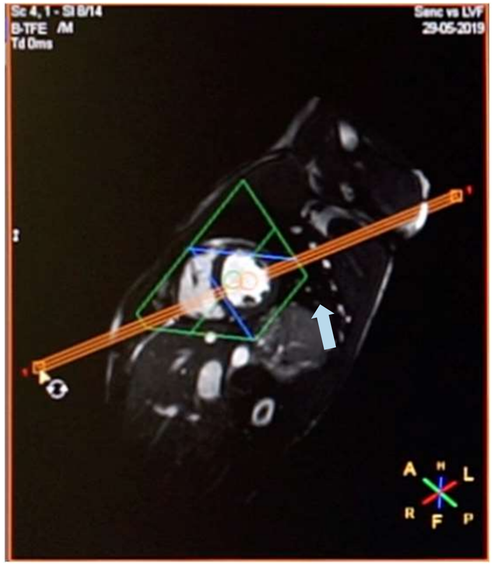


1. Double-click “4CH”-sequence on the left-hand panel. On the “sSA rest wh” view, place the stack through the papillary muscle, intersecting the peak of the right ventricle.

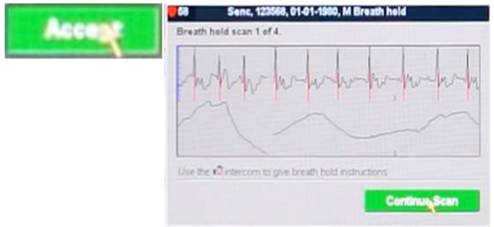


2. Click “Accept” and start the scan while the patient holds his breath, as described before.

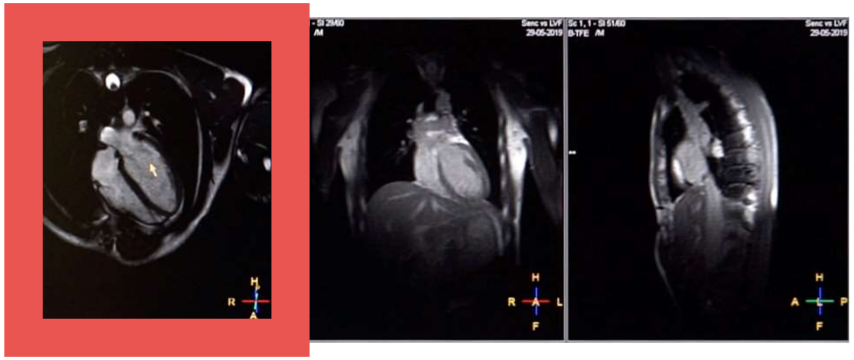

3. After scanning is finished, drag the 4-chamber view into the left-hand field.

# 1.6 2-chamber view (2CH)

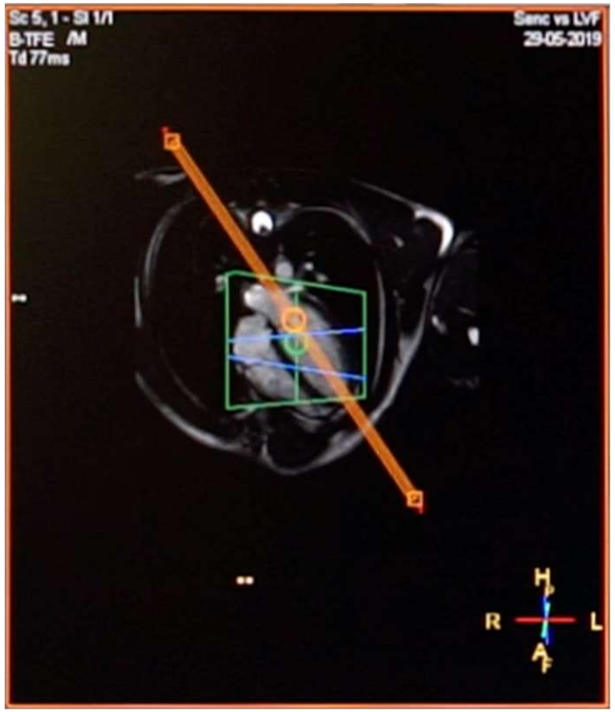


1. Double-click "2CH"-sequence on the left-hand panel. On the 4-chamber view, place the stack through the apex of the left ventricle and the center of the mitral valve.

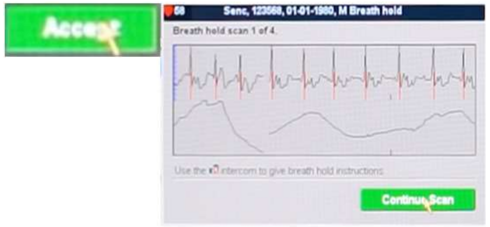


2. Click "Accept" and "Continue Scan" while the patient holds his breath.

# 1.7 3-chamber view (3CH)

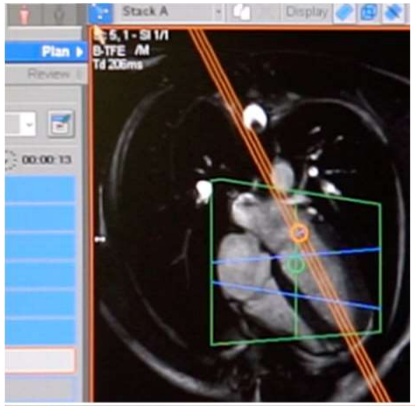


1. Double-click "3CH"-sequence on the left-hand panel. On the 4-chamber view, select the "3 point plan tool" from the upper panel.

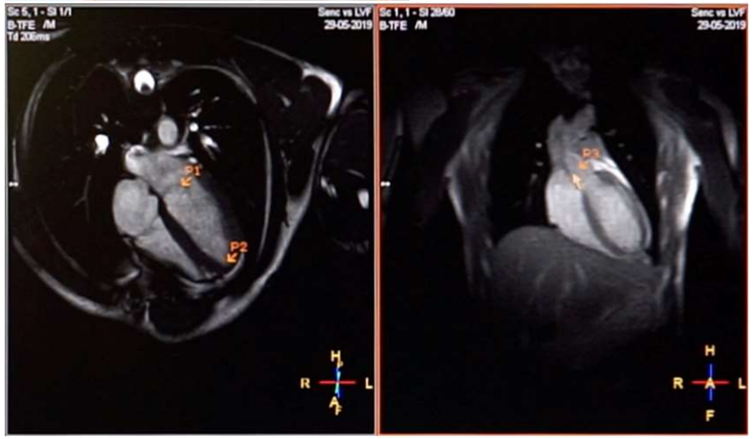


2. Place the first point into the center between the mitral valve leaflets, the second point in the apex (both in the 4-chamber view) , and the third point into the left-ventricular outflow tract (LVOT) of the coronal survey.

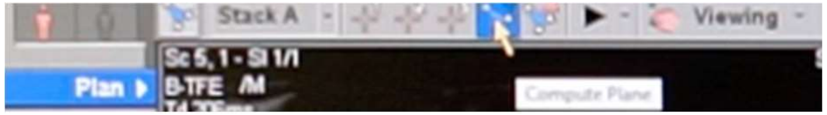


3. Select the "compute plane" tool from the upper panel to show the planned stack.

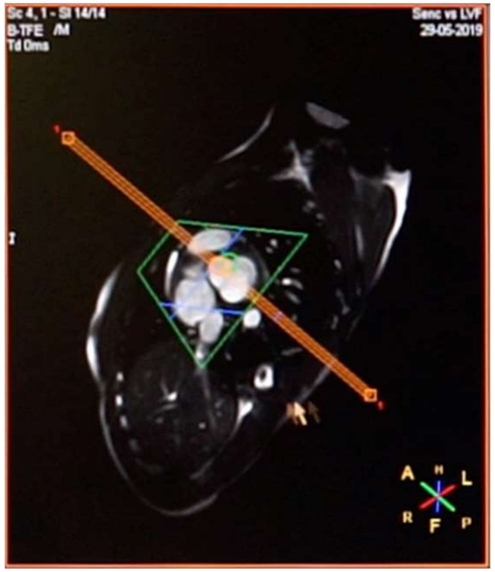

3. Control the orientation of the stack in the basal short-axis view. The stack should be placed directly in and parallel to the LVOT.

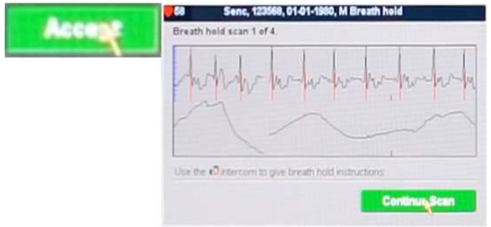


4. Click "Accept" and then "Continue Scan", while the patient holds his breath.

# 2.1 fSENC apical short-axis view

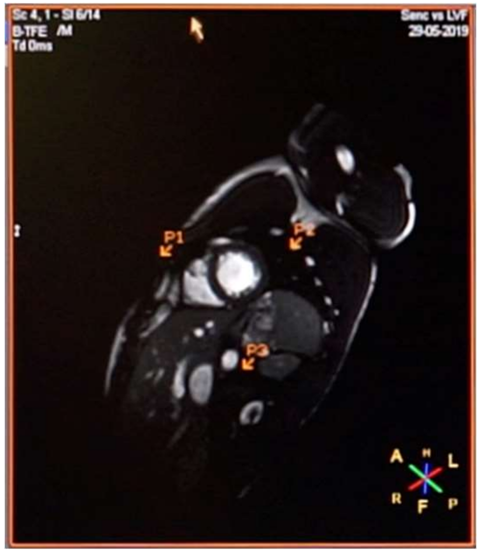

1. Double-click “fSENC SA a”-sequence on the left-hand panel. Drag the “sSA rest wh” images into the left-hand frame and select the apical slice. Use the “3-point plan tool”, as described in steps 1 and 3 of page 9 (3CH), to copy the geometry of the cine-SSFP images.

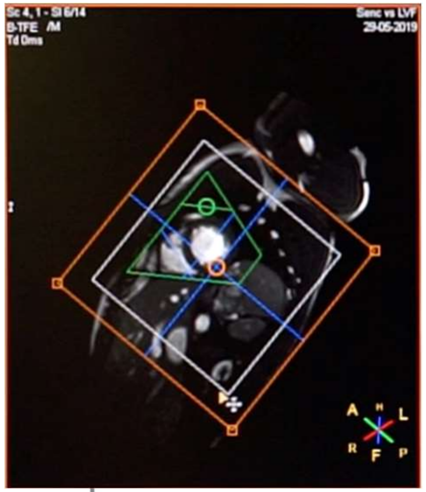

2. Position the white “SENC-box” with the heart in the center.

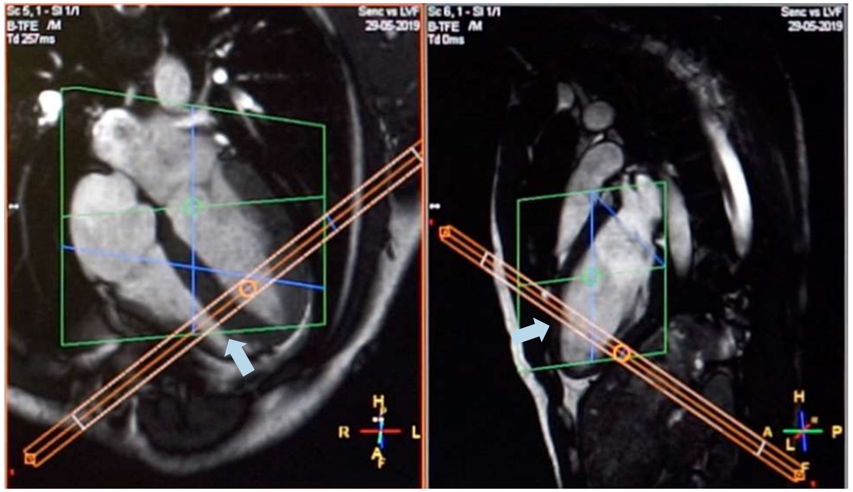

3. Using the 4CH view (dragged into the left frame) and the 2CH view (dragged into the middle frame), control the position of the stack. It should be perpendicular to the myocardium in both views (arrows).

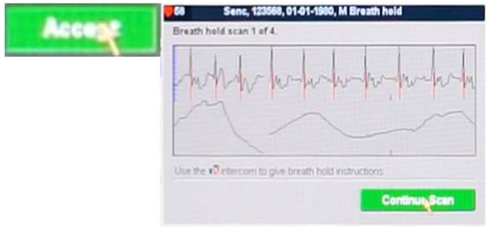


4. Click ”Accept” and then “Continue Scan”, while the patient continues breathing.

# 2.2 fSENC midventricular short-axis view

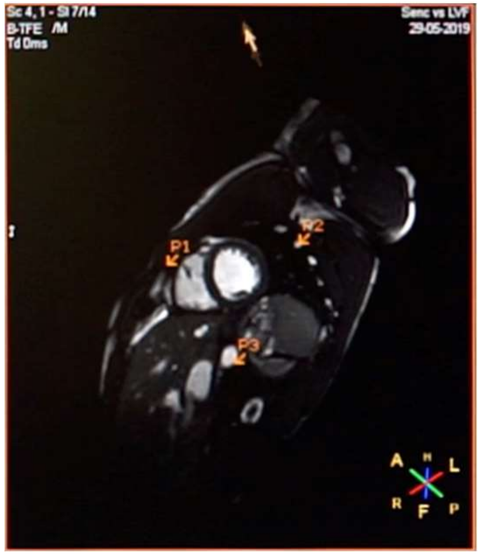


1. Double-click on “fSENC SA mid”-sequence on the left-hand panel. Drag the “sSA rest wh” images into the left-hand frame and select a midventricular slice. Use the “3-point plan tool” (described in steps 1 and 3 of page 9) to copy the geometry of the cine-SSFP images. Then position the SENC-box with the heart in the center (step 2 page 10).

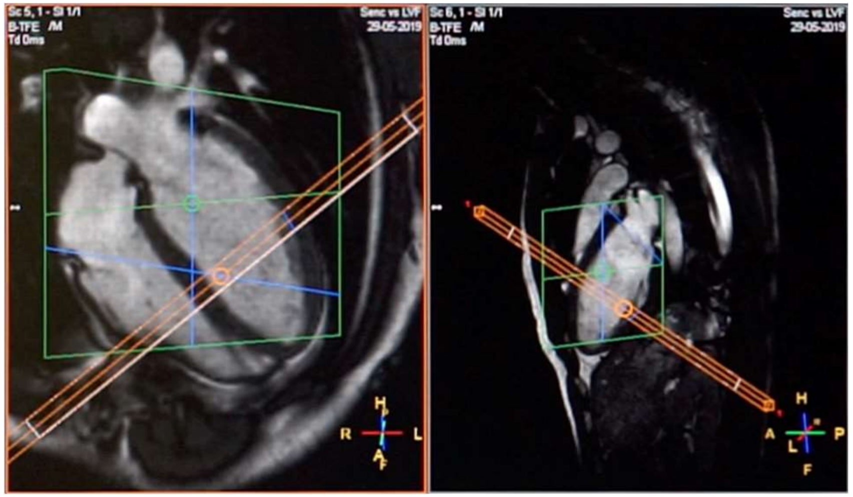

2. Using the 4CH view (dragged into the left frame) and the 2CH view (dragged into the middle frame), control the position of the stack. It should be perpendicular to the myocardium in both views.

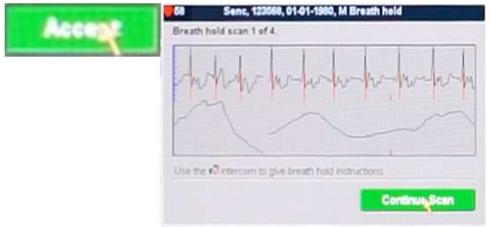


3. Click ”Accept” and then “Continue Scan”, while the patient continues breathing.

# 2.3 fSENC basal short-axis view

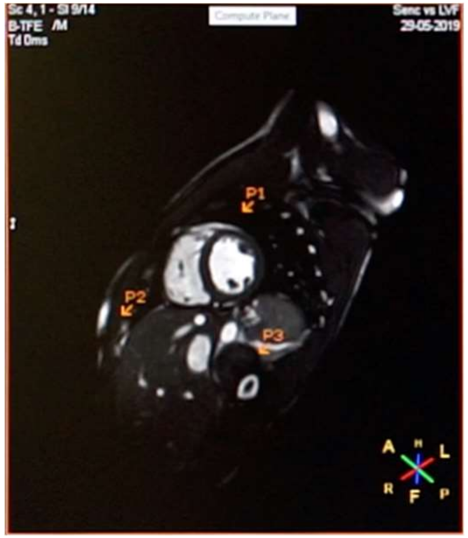


1. Double-click “fSENC SA bas”-sequence on the left-hand panel. Drag the “sSA rest wh” images into the left-hand frame and select a midventricular slice. Use the “3-point plan tool” as described in steps 1 and 3 of page 9 (3CH) to copy the geometry of the cine-SSFP images. Then position the SENC-box with the heart in the center (step 2 page 10).

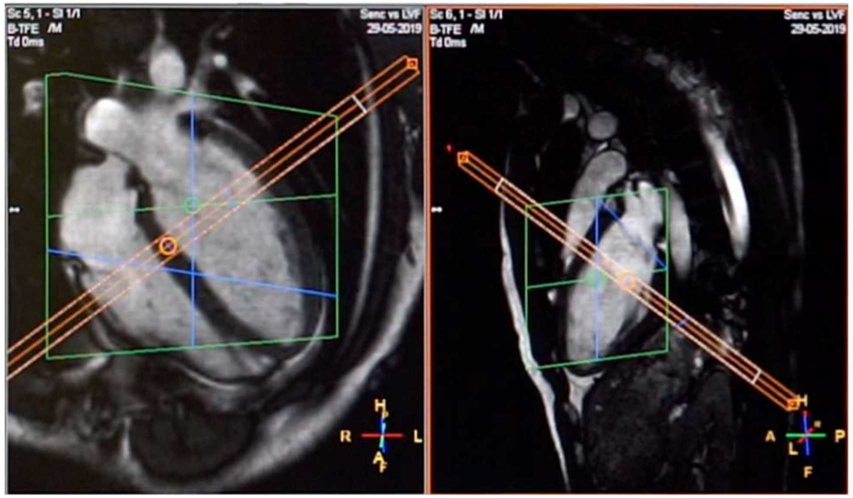

2. Using the 4CH view (dragged into the left frame) and the 2CH view (dragged into the middle frame), control the position of the stack. It should be perpendicular to the myocardium in both views.

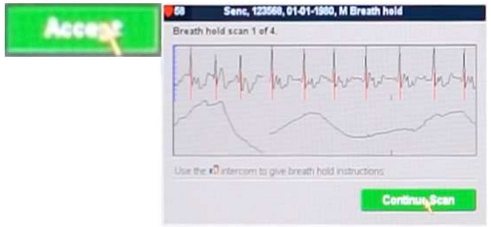


3. Click ”Accept” and then “Continue Scan”, while the patient continues breathing.

# 2.4 fSENC 4-chamber view

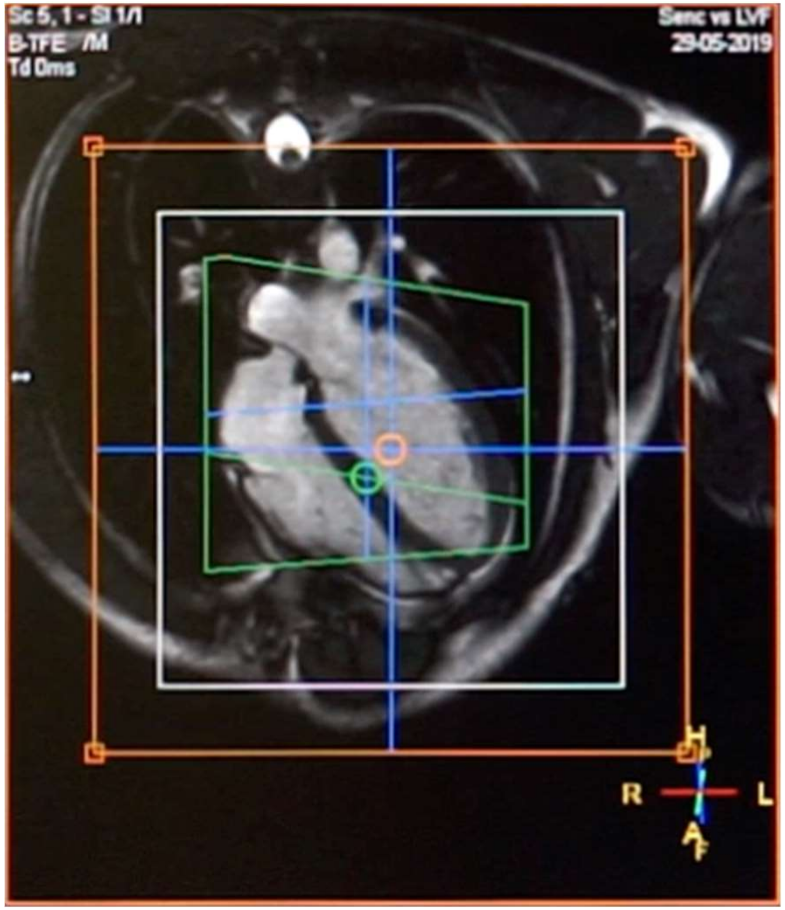


1. Select "fSENC rest 4ch"-sequence from the left-hand panel. Copy the geometry from the cine-SSFP 4-chamber view by dragging it into the image frame. Ensure, that the white SENC-Box is positioned with the heart in the center (step 2 page 10).

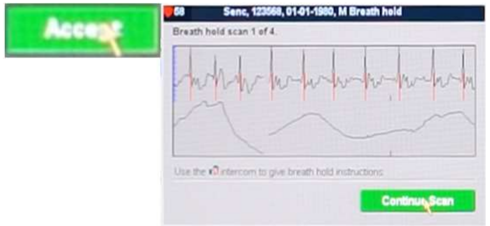


2. Click "Accept" and then "Continue Scan", while the patient continues breathing.

# 2.5 fSENC 2-chamber view

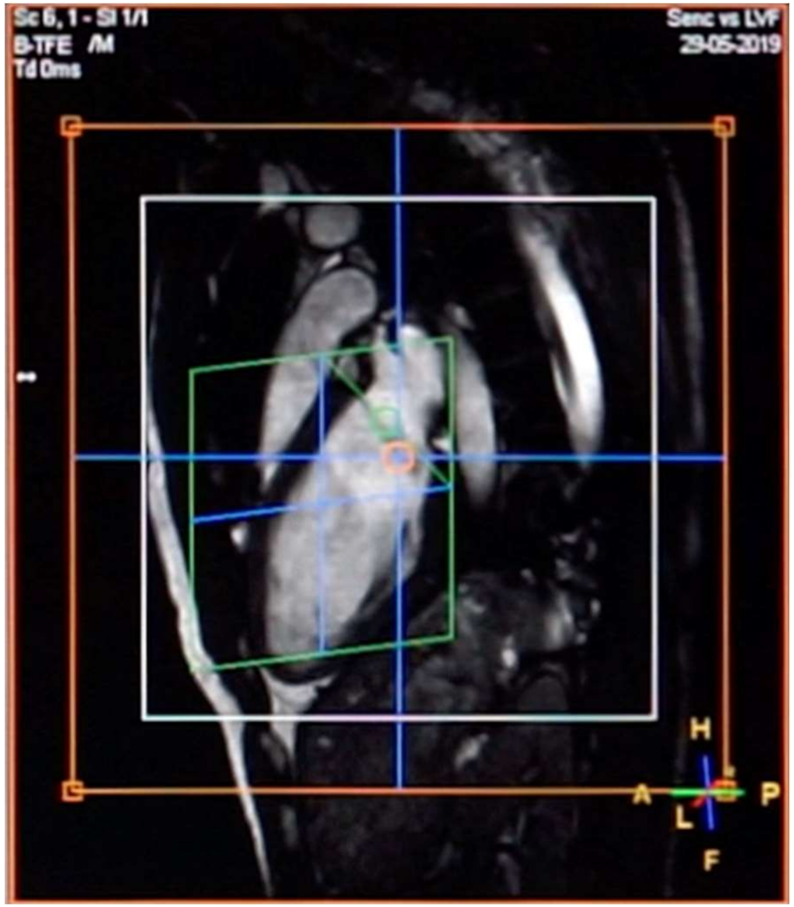


1. Select “fSENC rest 2ch”-sequence from the left-hand panel. Copy the geometry from the cine-SSFP 2-chamber view by dragging it into the image frame. Ensure, that the white SENC-Box is positioned with the heart in the center (step 2 page 10).

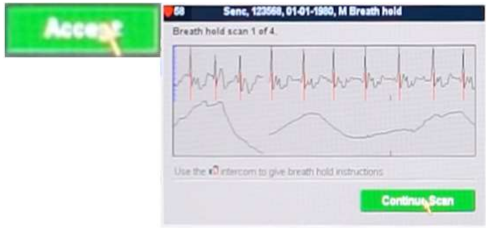


2. Click ”Accept” and then “Continue Scan” while the patient continues breathing.

# 2.6 fSENC 3-chamber view

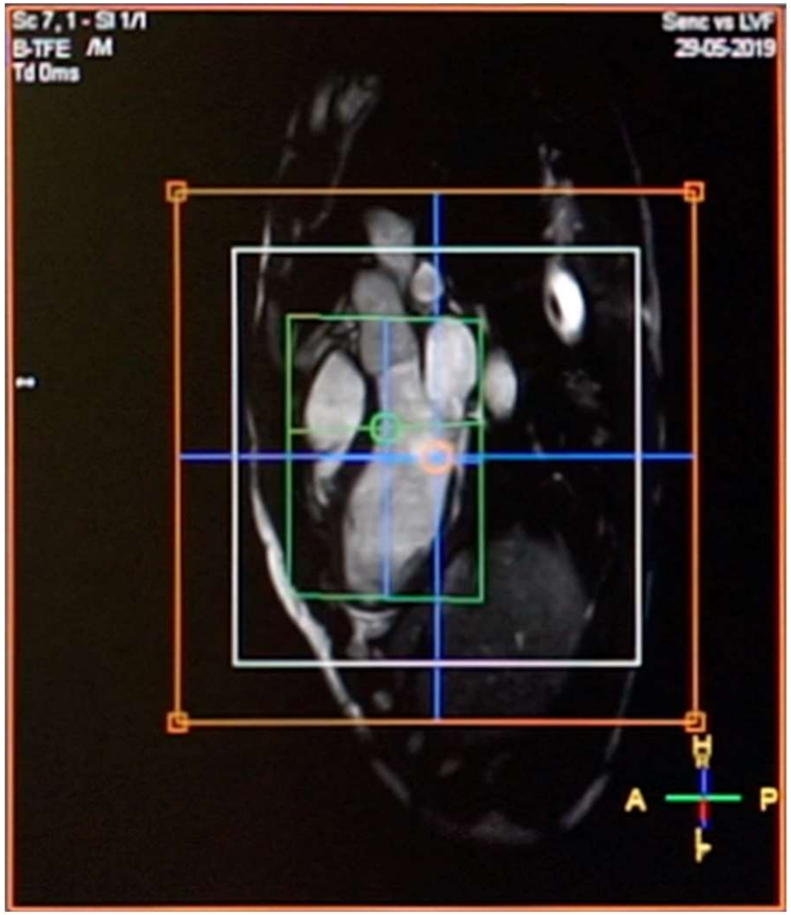


1. Select “fSENC rest 3ch”-sequence from the left-hand panel. Copy the geometry from the cine-SSFP 3-chamber view by dragging it into the image frame. Ensure, that the white SENC-Box is positioned with the heart in the center (step 2 page 10).

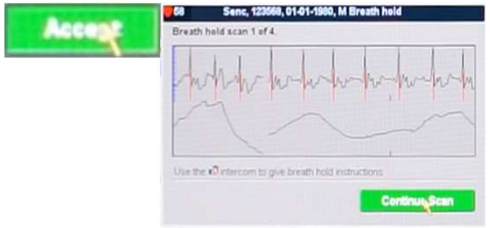


2. Click ”Accept” and then “Continue Scan” while the patient continues breathing.

# 3. Quality Check

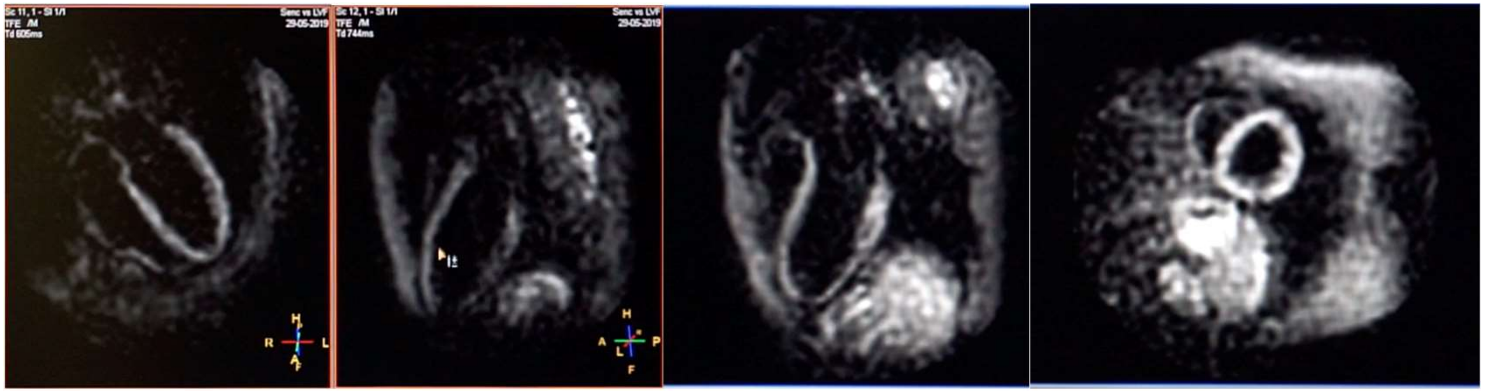

In case of disturbing artifacts, simply rotate the stack and repeat the scan. This is shown using the fSENC rest 4CH scan. Open the scan (double-click on the left-hand panel). Rotate the stack (orange square, see arrow) and repeat the scan.

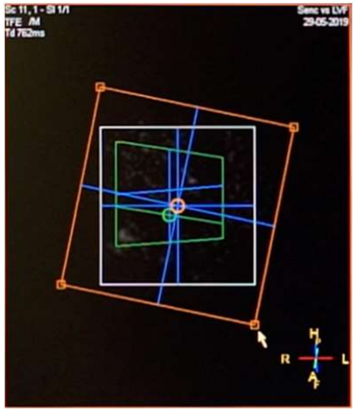

DEUTSCHES HERZZENTRUM DER CHARITÉ

UKE HAMBURG

We thank the whole media team of the Deutsches Herzzentrum der Charité around Christian Meier and Kornelius Glaser for the support regarding the recording and editing of the videos.

CHARITÉ

MRI-CORE-LAB BERLIN
