# Supplementary material for: How we scan cardiac anatomy and function using cardiovascular magnetic resonance: a practical video guide
Source: Eur Heart J Imaging Methods Pract. 2025 Jul 7;3(2):qyaf090. doi: 10.1093/ehjimp/qyaf090 (PMC12287925; doi:10.1093/ehjimp/qyaf090)
Supplement: qyaf090_Supplementary_Data [file qyaf090_supplementary_data.zip › Still Video 1.pdf]

CMR-acquisition of cardiac function using SSFP-cine & fSENC

English Version
